# Effect of various interventions on relieving non-coring needle puncture-related pain in patients with totally implantable venous access port: a network meta-analysis of randomized control trials

**DOI:** 10.3389/fsurg.2026.1839473

**Published:** 2026-06-26

**Authors:** Xiaojun Wang, Jing Tan, Hui Yuan, Juan Chen, Yingying Jiao

**Affiliations:** Department of Oncology, First Affiliated Hospital, Army Medical University, Chongqing, China

**Keywords:** network meta-analysis, non-coring needle, pain, randomized control trials, totally implantable venous access port

## Abstract

**Background:**

The totally implantable venous access port (TIVAP) is an infusion device that can be permanently implanted in the subcutaneous tissue. However, patients with a TIVAP often experience considerable pain during non-invasive needle puncture. Alleviating this procedure-related pain is therefore particularly important. This network meta-analysis compared the effectiveness of several common interventions for reducing pain during non-invasive needle puncture in patients with a TIVAP, aiming to provide evidence to support clinical practice.

**Methods:**

Randomized controlled trials investigating pain reduction during non-invasive needle puncture in patients with a TIVAP were included. Two researchers independently screened the literature and extracted data. Study quality was assessed using the Cochrane Risk of Bias 2.0 (RoB2) tool. The quality of evidence was evaluated with the CINeMA (Confidence in Network Meta-Analysis) framework. Data analyses were performed using Stata 14 software.

**Results:**

Seven trials involving nine interventions and 942 patients were included. The network meta-analysis showed that, compared with placebo, cryotherapy, cutaneous stimulation therapy, and standard care, lidocaine cream combined with the Valsalva maneuver was associated with lower pain scores. Lidocaine spray, lidocaine cream, and the Valsalva maneuver alone also yielded lower pain scores compared with standard care. Lidocaine cream reduced pain scores compared with cutaneous stimulation therapy. Based on surface under the cumulative ranking curve (SUCRA) probabilities, the interventions ranked as follows: lidocaine cream combing with valsalva maneuver (93.3%) > lidocaine spray (79.6%) > lidocaine cream (68.6%) > valsalva maneuver (66.5%) > cold spray (51.3%) > placebo (33.3%) > cryotherapy (33.1%) > cutaneous stimulation therapy (18.2%) > standard care (6.1%).

**Conclusions:**

Based on the current evidence, lidocaine cream combined with the Valsalva maneuver had the highest probability of being the most effective among the evaluated interventions. However, the included randomized controlled trials were limited in number, the evidence network was sparse, the methodological quality of some studies was suboptimal, and direct head-to-head comparisons between different interventions were lacking. This finding should be regarded as an exploratory result derived from limited evidence and interpreted with caution. Therefore, strong clinical recommendations cannot be made at present. Larger, rigorously designed, and adequately blinded randomized controlled trials are needed to directly compare the most promising interventions (e.g., lidocaine cream combined with the Valsalva maneuver vs. lidocaine spray) and to employ standardized pain assessment tools together with clinically meaningful pain relief thresholds, in order to further validate these findings and guide clinical practice.

## 1 Introduction

The totally implantable venous access port (TIVAP) is a subcutaneous infusion system that can be retained within the human body for an extended period (1).It provides patients with a long-term venous vascular channel (2). Because it is completely implanted, TIVAP not only reduces the pain associated with repeated intravenous infusions but also allows patients to move freely during chemotherapy, without interfering their daily lives and thereby improving their quality of life. It is particularly favored by patients with advanced tumors (3, 4). TIVAP puncture uses a non-coring needle, which has a specialized tip design. This needle penetrates the skin more slowly than a conventional puncture needle, resulting in more pronounced and longer-lasting pain. The puncture must be repeated regularly (every 7 days during treatment and every 28 days for maintenance during non-treatment periods), giving the pain a repetitive and cumulative psychological impact. Unlike other venipunctures, TIVAP puncture requires vertical insertion into the subcutaneously implanted port septum, with specific angle and depth requirements, and may involve multi-layer stimulation of the skin, subcutaneous tissue, and silicone septum. Relevant studies have shown that the incidence of mild and moderate pain caused by the insertion of the TIVAP needle is 67.5% and 34.9%, respectively, with a mean pain score of 3.91 ± 1.35 (5, 6). Compared with other venous access devices, repeated TIVAP needle insertion can cause fear and anxiety in patients (7, 8). Pain can also increase anxiety, trigger a physiological stress response, and may even lead patients to avoid the procedure (7, 9). Furthermore, the pain can cause patients to shy away from the injection site, resulting in improper needle insertion and potentially leading to serious complications such as extravasation of chemotherapy drugs (10). Therefore, reducing and alleviating the pain caused by non-coring needle puncture has attracted widespread attention in the academic community.

Various interventions, both pharmacological and non-pharmacological, have been proposed and applied in clinical practice to address pain during non-coring needle puncture in patients with TIVAP. These include cryotherapy, cutaneous stimulation therapy, cold spray, lidocaine cream, and others. Lidocaine cream (e.g., EMLA) is a eutectic mixture of 2.5% lidocaine and 2.5% prilocaine. It achieves a superficial anesthetic effect by accumulating at sensory nerve endings in the dermis, effectively relieving venipuncture pain (11, 12). Although widely used in clinical practice, its onset of action is relatively slow, taking approximately 60–120 min (11, 13). The main component of cold spray is pentafluoropropane. When applied locally, it produces an instantaneous cooling effect, slows peripheral nerve conduction, blocks pain impulse transmission, and reduces the patient's pain. Cold spray can be used for procedures such as intravenous catheterization, breast biopsy, and minor surgical operations, providing rapid and effective pain relief (14–16). Lidocaine spray combines the analgesic effect of lidocaine cream with the rapid onset of cold spray, offering the advantages of quick onset, prolonged anesthesia, and ease of use. It is currently used for pain during venous and arterial punctures, chest tube removal, and intrauterine device placement (11, 17, 18).

However, current evidence on the effectiveness and comparative efficacy of these interventions remains limited, and a systematic evaluation is lacking. Most existing studies are randomized controlled trials with pairwise comparisons, lacking a comprehensive comparison of the relative efficacy of different interventions. Traditional pairwise meta-analysis can only compare two interventions and cannot determine the hierarchy of multiple interventions in the absence of direct comparative evidence. Network meta-analysis (NMA) can integrate both direct and indirect evidence, provide a ranking of the relative efficacy of interventions, and thus guide clinical decision-making more comprehensively. Although these interventions differ in form, they are all applied to the same target population at the same procedural stage for pain relief. Most of them alleviate pain by inhibiting nerve conduction, modulating local tissue tension, or providing distraction, and share similar outcome measures. This study aimed to conduct a network meta-analysis to systematically compare the relative efficacy of nine interventions—pharmacological (lidocaine cream, lidocaine spray, cold spray), non-pharmacological (Valsalva maneuver, cryotherapy, cutaneous stimulation therapy), and a combination intervention (lidocaine cream plus the Valsalva maneuver)—relative to standard care or placebo, in reducing non-coring needle puncture-related pain in oncology patients with TIVAP, and to provide an optimal intervention ranking to inform clinical practice.

## 2 Methods

This NMA was performed following the Preferred Reporting Items for Systematic Reviews and Meta-Analyses extension statement for network meta-analysis (PRISMA-NMA) guidelines.

### 2.1 Inclusion criteria

Studies were included if they met the following criteria:

P (Participants): 1) Patients diagnosed with a tumor who had a TIVAP implanted. 2) Patients with an indwelling TIVAP who required non-coring needle puncture (for chemotherapy administration or routine maintenance, e.g., needle replacement every 7 days and port access every 28 days; including both first-time and maintenance punctures). 3) No restrictions on sex or age.

I (Interventions): Included but not limited to lidocaine cream, lidocaine spray, cold spray, cryotherapy, cutaneous stimulation therapy, Valsalva maneuver, and combination regimens; comparators included standard care, placebo, etc.

Lidocaine cream: a eutectic mixture of 2.5% lidocaine and 2.5% prilocaine, applied at approximately 1.0–1.5 g/10 cm^2^ to the puncture site, covered with a transparent dressing, and left in place for 30–120 min before being wiped off prior to puncture. 2) Lidocaine spray: the main ingredient is lidocaine; approximately 10 cm from the puncture site, 3 sprays are delivered, repeated once (2 applications total, 1–2 min apart, total dose approximately 27 mg); puncture is performed 1–2 min later. 3) Cold spray: the main ingredient is pentafluoropropane; after skin disinfection, the spray is applied 10 cm from the site, pressed twice, each time for 5 s until the skin turns white; puncture must be completed within 30 s. 4) Cryotherapy: a silica gel cold pack frozen for 3 h is applied to the puncture site 10 min before puncture for approximately 3 min. 5) Cutaneous stimulation therapy: firm, slow, circular massage is applied with the fingertips over an area of approximately 12 cm in diameter, at least 4 cm away from the puncture site, for at least 2 min. 6) Lidocaine cream combined with the Valsalva maneuver: lidocaine cream is applied 1 h in advance, and the patient performs the Valsalva maneuver (deep inspiration followed by forced expiration against a closed glottis for 5–10 s) during puncture. 7) Valsalva maneuver alone: only the breath-holding maneuver described above is performed during puncture. 8) Placebo: medical white petrolatum (cream placebo) with an identical appearance or mineral water spray (spray placebo), administered in the same manner as the corresponding active intervention. 9) Standard care: direct puncture after routine disinfection without any additional analgesic intervention.

O (Outcomes): Pain was assessed using tools including the Visual Analog Scale, Numerical Rating Scale, Faces Pain Scale, and Pain Behavior Checklist. In all studies, pain was evaluated immediately after puncture completion, either by patient self-report or nurse observation/recording.

S (Study design): Only randomized controlled trials (RCTs) were included; publication languages were restricted to Chinese and English.

### 2.2 Exclusion criteria

1. Duplicate publications;
2. Studies from which data could not be extracted;
3. Cross-over designs, cluster randomized trials, quasi-randomized trials, and before-after self-controlled designs.

### 2.3 Search strategy

Pubmed, the Cochrane Central Register of Controlled Trials, Embase, Chinese Biomedical Literature Database, China National Knowledge Infrastructure and CINAHL databases were searched from inception to March 2026. We included RCTs that investigated pain reduction during non-coring needle puncture in patients with TIVAP. The search combined subject headings and free-text terms. Search terms included: totally implantable venous access port, lidocaine cream, standard care, Valsalva maneuver, placebo, cold spray, lidocaine spray,etc. The detailed search strategy is provided in the supplementary materials (Supplementary Table S1).

### 2.4 Literature screening and data extraction

All retrieved citations were imported into Endnote. After removing duplicates, two researchers independently screened the literature and extracted the data. First, titles, keywords, and abstracts were screened according to the inclusion and exclusion criteria. The full texts of the initially eligible articles were then downloaded and reviewed, and the final included studies were selected. Data were extracted into a standardized Excel spreadsheet. Extracted information included: first author, year of publication, trial period, sample size, interventions, outcomes, etc. After extraction, each item was cross-checked. Any discrepancies were resolved by re-examining the original full text and re-extracting the data.

### 2.5 Quality assessment

The Cochrane Risk of Bias 2.0 (RoB2) tool was used to assess the risk of bias in the included RCTs. RoB2 covers five domains: bias arising from the randomization process, bias due to deviations from intended interventions, bias due to missing outcome data, bias in measurement of the outcome, and bias in the selection of the reported result. Each domain contains several signaling questions. RoB2 provides an algorithm that yields a risk-of-bias judgment of “low risk of bias,” “some concerns,” or “high risk of bias” for each domain and an overall assessment. The tool automatically generates a domain-level and overall risk-of-bias assessment based on the responses; assessors may decide whether to adopt the algorithm-generated judgment after considering the specific context. As the outcome of interest in this review was pain score, the risk-of-bias assessment focused on this outcome. Two reviewers independently evaluated each included study using the RoB2 tool. Disagreements were resolved through discussion; if consensus could not be reached, a third reviewer arbitrated.

### 2.6 Statistical analysis

All analyses were performed using a network suite of commands in Stata 14.0. The network package performed the network meta-analysis under a frequentist framework (19). Heterogeneity was assessed using the I^2^ statistic. If I^2^ ≤ 50%, indicating low heterogeneity, a fixed-effect model was considered (20); otherwise, a random-effects model was chosen (21). Given the anticipated clinical and methodological heterogeneity, a random-effects model was adopted for the network meta-analysis. Consistency was evaluated using node-splitting analysis to compare direct and indirect estimates. If *P* > 0.05, a consistency model was adopted for the network Meta-analysis and the results were ranked. If *P* ≤ 0.05, an inconsistency model was used and the sources of inconsistency were explored. The outcomes were ranked using the surface under the cumulative ranking curve (SUCRA), with larger areas indicating a better intervention effect. Comparison-adjusted funnel plots were used to assess small-study effects and potential publication bias across interventions.

### 2.7 Evaluation of evidence quality

The Confidence in Network Meta-Analysis (CINeMA) framework was applied to evaluate the quality of evidence for the NMA results. This framework considers the NMA as a whole, accounting for the contribution of both direct and indirect evidence to the NMA estimates, and depicts a contribution matrix across six domains that may affect confidence: within-study bias, publication bias, indirectness, imprecision, heterogeneity, and incoherence. Confidence may be downgraded based on the severity of concerns in each domain, thereby systematically assessing their impact on the results. The final NMA evidence quality is graded as high, moderate, low, or very low.

## 3 Results

### 3.1 Literature search and included studies

**Figure 1:**
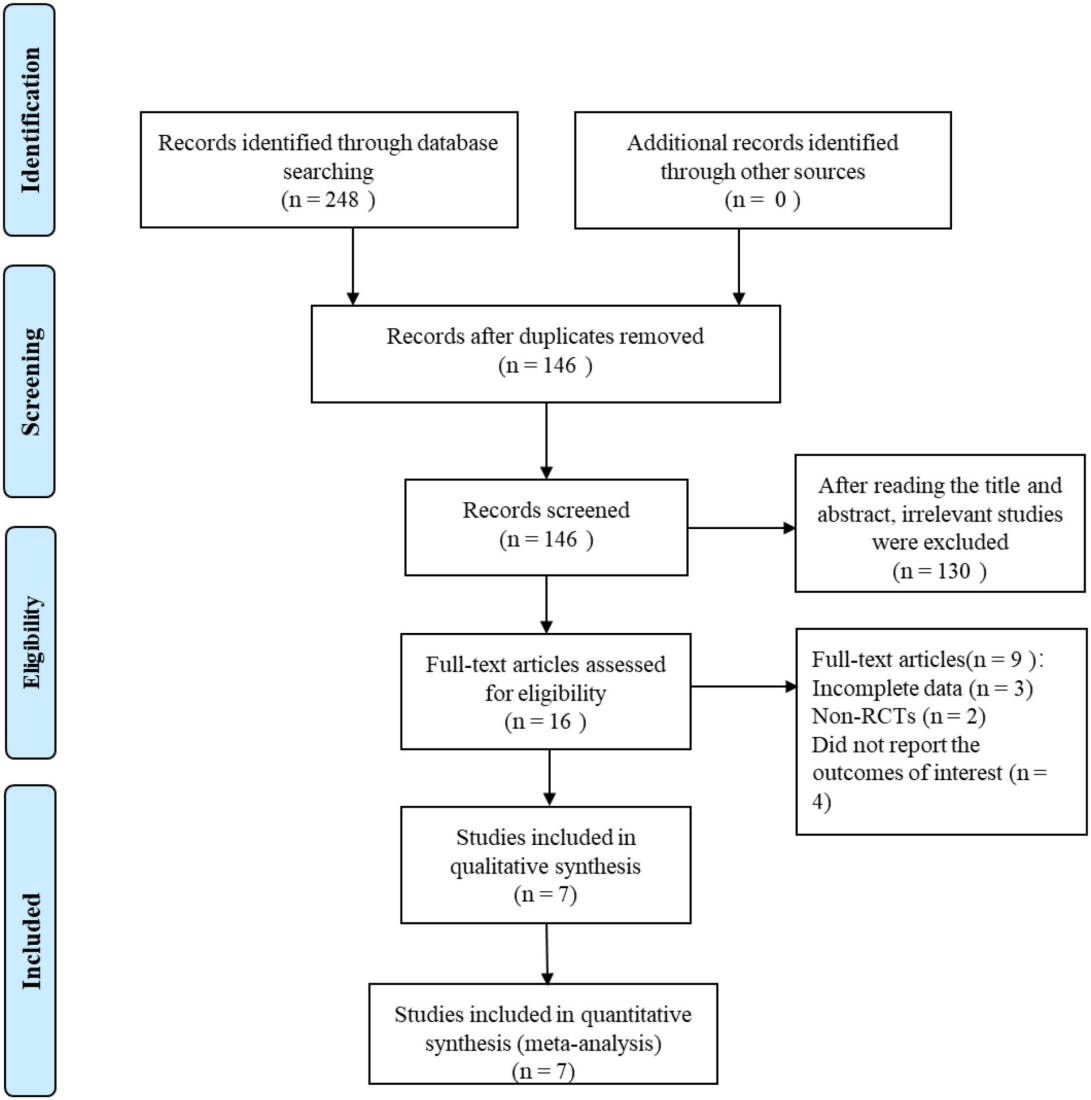
Flow Diagram of the literature selection process.

A total of 248 relevant articles were initially retrieved. After removing duplicates using Endnote, 146 articles remained. Following title and abstract screening and elimination of obviously irrelevant records, 16 articles were retained. After a thorough full-text review, seven RCTs (6, 10, 22–26) were ultimately included (Figure 1). These trials were published between 2015 and 2023 and enrolled a total of 942 patients. The interventions comprised lidocaine cream, lidocaine spray, cold spray, cryotherapy, cutaneous stimulation therapy, lidocaine cream combined with Valsalva maneuver, placebo, standard care, and Valsalva maneuver (Supplementary Table S2). The basic characteristics of the included studies are presented in Table 1.

**Table 1:** Characteristics of the included studies.

Study	Study design	Country	Date Collection Time	No. of patients	Age	Tumor type	Location of the infusion port	Puncture type	The specifications of the puncture needle	Pain assessment tool	Sources of funding	Conflict of interest	Intervention
Liu 2018 (22)	RCT	China	From January 2016 to December 2016	18	14–75	Breast cancer, lymphoma, leukemia, digestive tract tumors, etc.	NR	Maintenance period puncture	22G (Bard, USA)	Numerical Rating Scale	NR	NO	Lidocaine cream
				18	14–75								Standard care
Shi 2023	RCT	China	From April to May 202	53	55.40 ± 12.31	Breast cancer, gastrointestinal cancer, etc.	Chest	Maintenance period puncture	Bard	Numerical Rating Scale	Guangdong Nurses Association Project	NO	Lidocaine cream
				55	51.16 ± 10.24								Placebo
				51	55.65 ± 9.74								Valsalva maneuver
				64	54.09 ± 11.05								Lidocaine cream combing with valsalva maneuver
Li 2022 (10)	RCT	China	From May 2021 to June 2021	29	58.3 ± 10.8	NR	NR	Maintenance period puncture	20G (Bayer Germany)	Numerical Rating Scale	National Natural Science Foundation of China	NO	Lidocaine cream
				29	55.8 ± 10.4								Cold spray
Zhang 2019 (25)	RCT	China	From February 2017 to February 2018	30	53.68 ± 11.69	NR	NR	Maintenance period puncture	NR	Wong-Baker faces pain scale revision	NR	NO	Lidocaine cream
				30	53.68 ± 11.69								Cold spray
Zhu 2023 (26)	RCT	China	From January 2023 to March 2023	42	57.31 ± 12.17	Breast, gastrointestinal, respiratory, gynecological tumors	Chest	Maintenance period puncture	20G (Bayer Germany)	Visual Analogue Scale	The distinctive projects of Shanghai First People's Hospital	NO	Lidocaine spray
				42	56.05 ± 13.86								Placebo
Shin 2020	RCT	Korea	From May 2015 to May 2016	30	60.13 ± 12.54	Gastric cancer, colorectal cancer, lung cancer, etc.	Chest	Pre-treatment puncture before conventional chemotherapy	20G Huber puncture needle	Visual Analogue Scale	NR	NO	Lidocaine cream
				30	61.23 ± 11.47								Cryotherapy
				30	61.83 ± 12.43								Standard care
				30	60.43 ± 9.13								Cutaneous stimulation therapy
Yin 2018 (24)	RCT	China	From June 2016 to December 2016	228	NR	NR	NR	Including the initial and subsequent punctures	22G (BARD)	Numerical Rating Scale	NR	NO	Lidocaine cream
				133	NR								Placebo

RCT, randomized controlled trial; NR, not reported. .

### 3.2 Results of risk of bias

**Figure 2:**
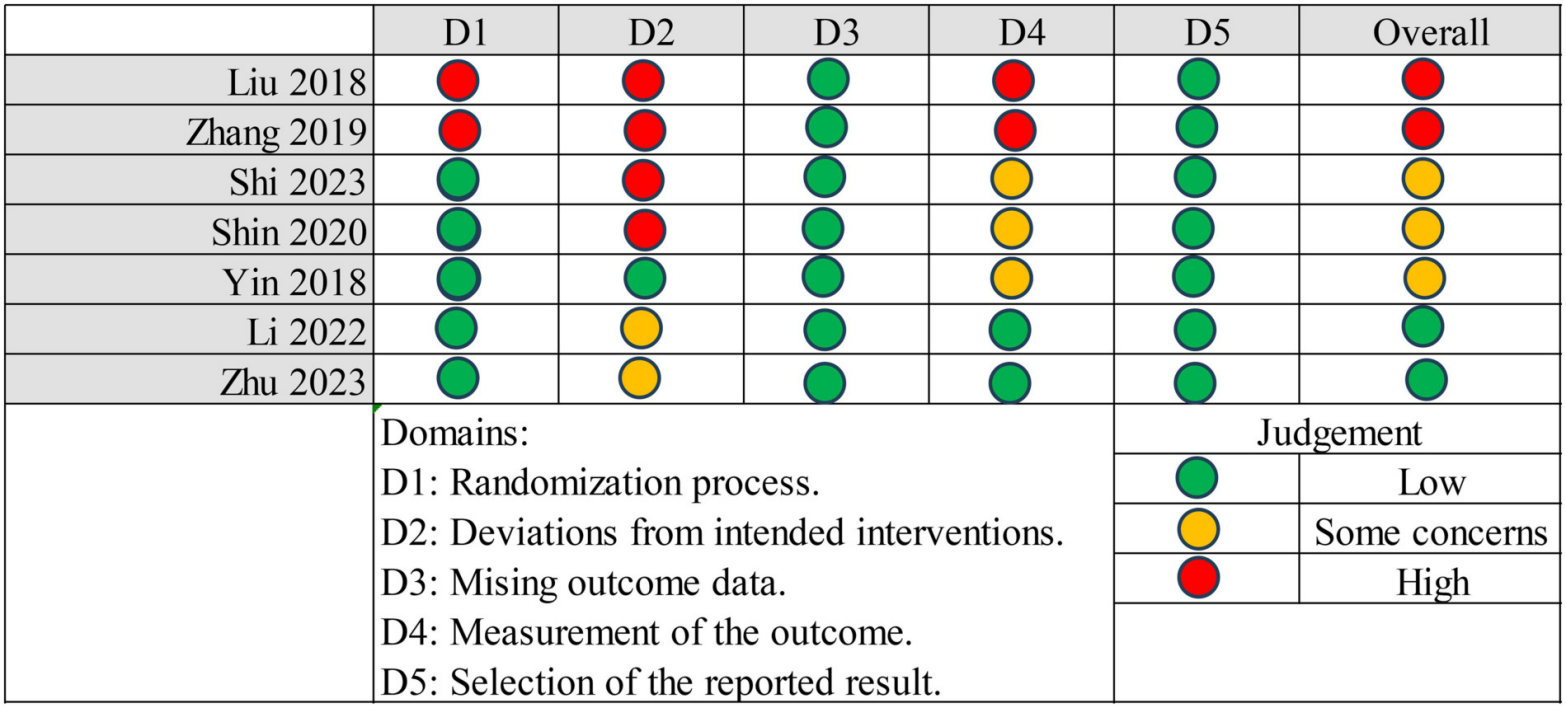
Results of risk of bias analysis.

Two studies (22, 25) were rated as having a high risk of bias, primarily because the randomization method, allocation concealment, and blinding were not described, and outcome assessment was unblinded. Three studies (6, 23, 24) were judged to raise some concerns, mainly due to inadequate blinding or unclear allocation concealment, although the overall study design was acceptable. Two studies (10, 26) were assessed as having a low risk of bias; these trials employed adequate randomization, clear allocation concealment, and blinded or objective self-reported outcome assessment, with standardized data handling. The results of the risk-of-bias assessment are shown in Figure 2.

### 3.3 Inconsistency test

**Figure 3:**
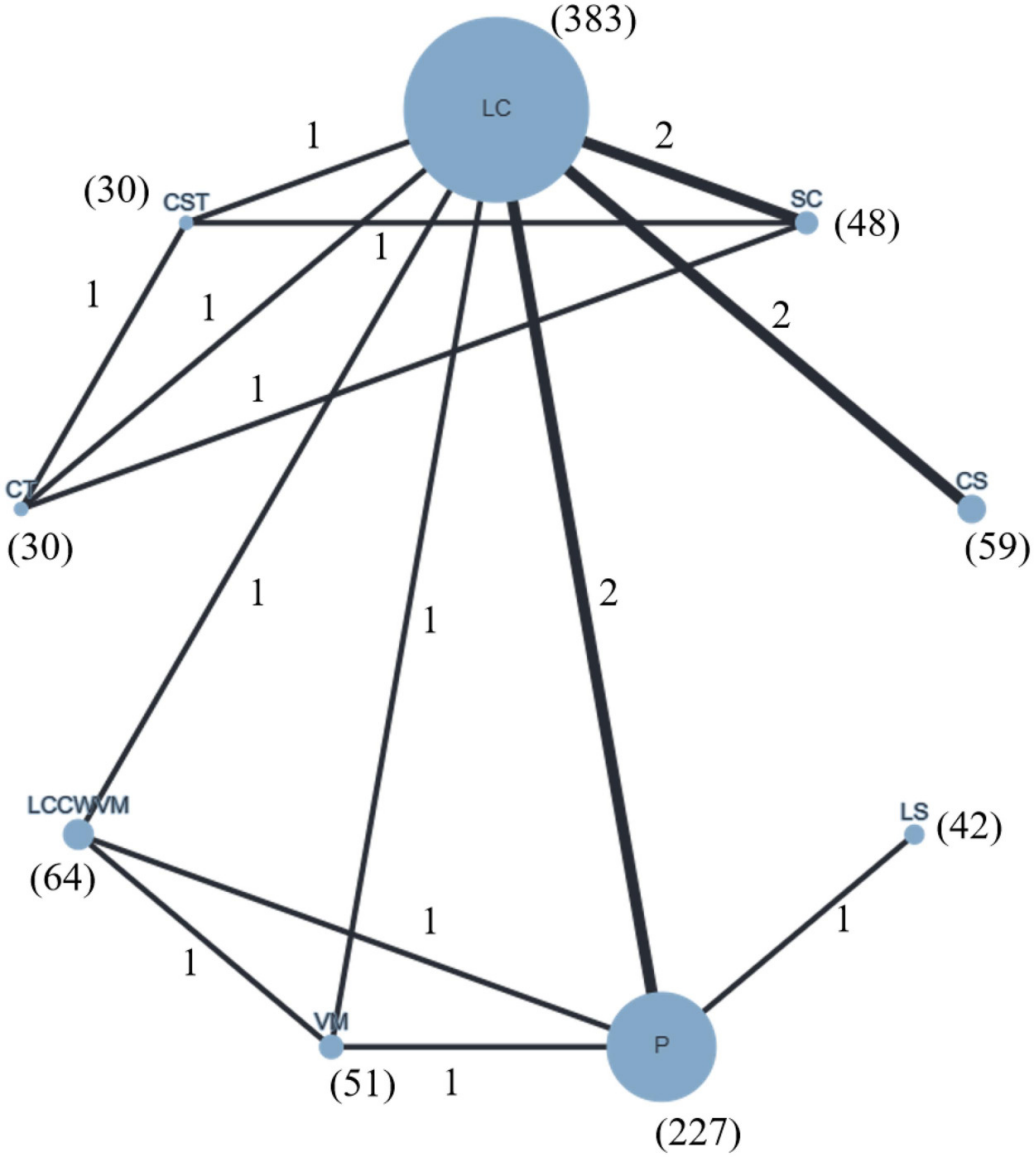
Evidence network relationship. SUCRA, surface under the cumulative ranking; LC, lidocaine cream; lidocaine spray; CS, cold spray; CT, cryotherapy; CST, cutaneous stimulation therapy; LCCWVM, lidocaine cream combing with valsalva maneuver; P, placebo; SC, standard care; VM, valsalva maneuver.

The overall network of intervention comparisons is displayed in Figure 3. Nodes represent the interventions, and the thickness of the connecting lines reflects the sample size for each direct comparison: thicker lines indicate more direct evidence, thinner lines indicate less, and absent lines indicate no direct comparison. Indirect comparisons can be made through the network meta-analysis. Because the network evidence plot contained closed loops, the node-splitting method was used to test for inconsistency. No statistically significant differences were found for any of the comparisons (*P* > 0.05), indicating that the direct and indirect evidence were consistent, and the data could be analyzed under the consistency model.

### 3.4 Results of a network meta-analysis

**Figure 4:**
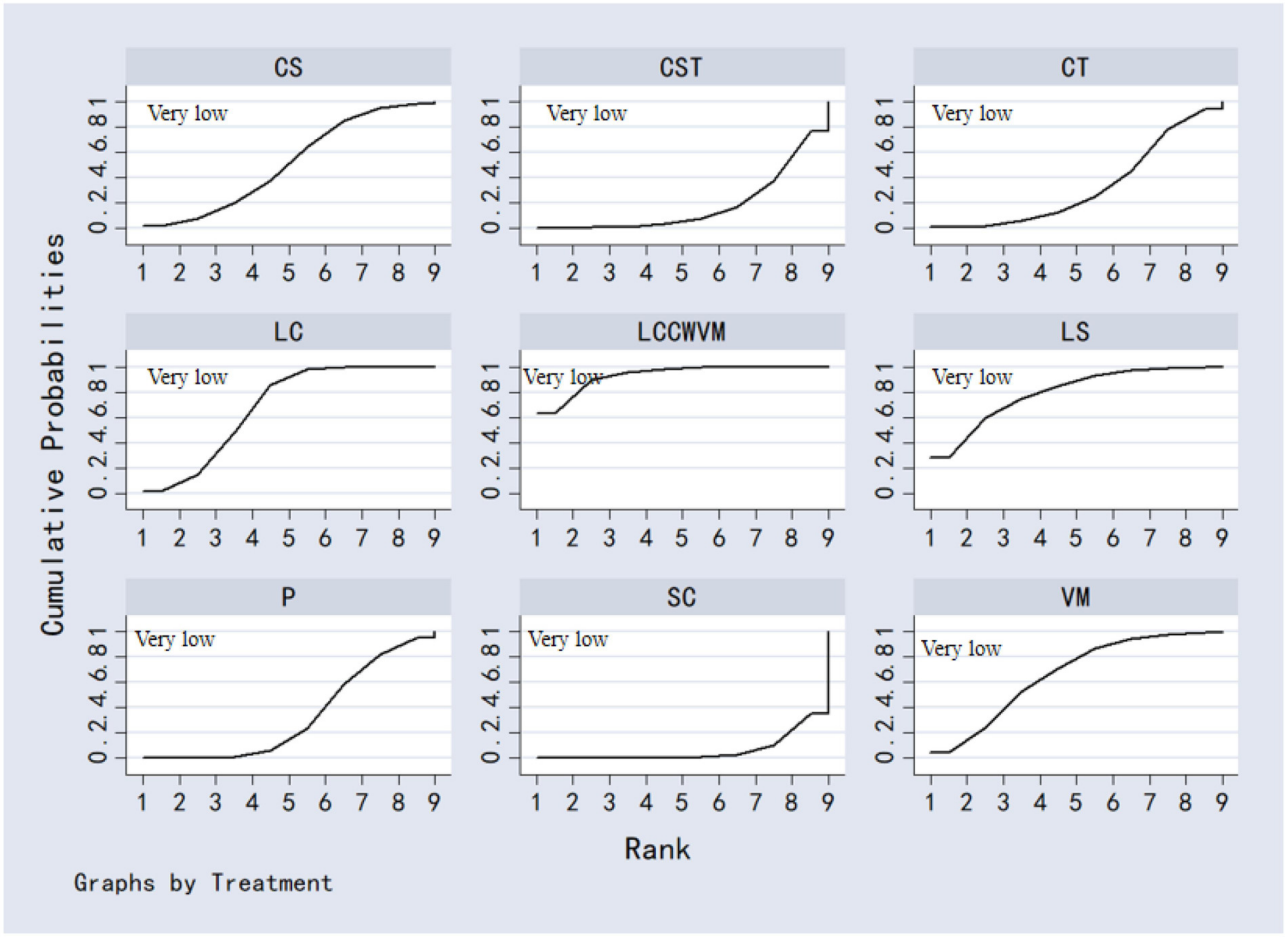
The SUCRA of the pain score. SUCRA, surface under the cumulative ranking; LC, lidocaine cream; S, lidocaine spray; CS, cold spray; CT, cryotherapy; CST, cutaneous stimulation therapy; LCCWVM, lidocaine cream combing with valsalva maneuver; P, placebo; SC, standard care; VM, valsalva maneuver.

The network meta-analysis showed that compared with placebo [SMD = −2.02, 95%CI (−3.43,−0.61)], cryotherapy [SMD = −2.17, 95%CI(−4.19,−0.15)], cutaneous stimulation therapy [SMD = −2.67, 95%CI(−4.70,−0.65)] and standard care [SMD = −3.15, 95%CI(−4.96,−1.33)], lidocaine cream combing with Valsalva maneuver significantly reduced patients' pain scores. Compared with standard care, lidocaine spray [SMD = −2.63, 95%CI(−4.81,−0.44)], lidocaine cream [SMD = −2.06, 95%CI(−3.21,−0.92)] and Valsalva maneuver [SMD = −2.11, 95%CI(−3.92,−0.30)] also significantly reduced pain scores. Compared with cutaneous stimulation therapy, lidocaine cream reduced the pain scores of patients [SMD = −1.59, 95%CI(−3.04,−0.13)]. No statistically significant differences were observed for the remaining pairwise comparisons. The network meta-analysis results are summarized in Table 2. A larger SUCRA value indicates a better pain-relieving effect. The SUCRA probability ranking was as follows: lidocaine cream combing with the Valsalva maneuver (93.3%) > lidocaine spray (79.6%) > lidocaine cream (68.6%) > Valsalva maneuver (66.5%) > cold spray (51.3%) > placebo (33.3%) > cryotherapy (33.1%) > cutaneous stimulation therapy (18.2%) > standard care (6.1%).The most notable finding was that lidocaine cream combined with the Valsalva maneuver ranked as the most effective intervention, suggesting that this combination is the most likely optimal approach (Figure 4, Table 3).

**Table 2:** The Results of network meta-analysis.

LCCWVM								
−0.52 (−2.60,1.56)	LS							
−1.09 (−2.49,0.32)	−0.56 (−2.43,1.30)	LC						
−1.04 (−2.54,0.47)	−0.52 (−2.60,1.57)	0.05 (−1.36,1.46)	VM					
−1.51 (−3.30,0.27)	−0.99 (−3.15,1.17)	−0.43 (−1.53,0.67)	−0.48 (−2.26,1.31)	CS				
**−2.02 (−3.43,−0.61)**	−1.50 (−3.04,0.03)	−0.94 (−1.99,0.12)	−0.99 (−2.39,0.42)	−0.51 (−2.03,1.01)	P			
**−2.17 (−4.19,−0.15)**	−1.65 (−4.01,0.71)	−1.08 (−2.53,0.37)	−1.13 (−3.15,0.89)	−0.65 (−2.47,1.17)	−0.14 (−1.94,1.65)	CT		
**−2.67 (−4.70,−0.65)**	−2.15 (−4.52,0.21)	**−1.59 (−3.04,−0.13)**	−1.64 (−3.66,0.39)	−1.16 (−2.99,0.66)	−0.65 (−2.45,1.15)	−0.51 (−2.05,1.03)	CST	
**−3.15 (−4.96,−1.33)**	**−2.63 (−4.81,−0.44)**	**−2.06 (−3.21,−0.92)**	**−2.11 (−3.92,−0.30)**	**−1.63 (−3.22,−0.05)**	−1.12 (−2.68,0.43)	−0.98 (−2.43,0.47)	−0.47 (−1.92,0.98)	SC

LC, lidocaine cream; LS, lidocaine spray; CS, cold spray; CT, cryotherapy; CST, cutaneous stimulation therapy; LCCWVM, lidocaine cream combing with valsalva maneuver; P, placebo; SC, standard care; VM, valsalva maneuver. Bold font indicates that the difference was statistically significant; Negative values indicated that the pain score of the row intervention was lower than that of the column intervention; The ranking equivalent to clinical superiority. Moreover, the confidence intervals of some comparisons were relatively wide, and therefore should be interpreted with caution.

**Table 3:** The Rankings of each interventions.

Intervention	SUCRA	PrBest	MeanRank
CS	51.3	1.8	4.9
CST	18.2	0.1	7.5
CT	33.1	0.6	6.4
LC	68.6	1.7	3.5
LCCWVM	93.3	62.8	1.5
LS	79.6	28.4	2.6
P	33.3	0	6.3
SC	6.1	0	8.5
VM	66.5	4.6	3.7

LC, lidocaine cream; LS, lidocaine spray; CS, cold spray; CT, cryotherapy; CST, cutaneous stimulation therapy; LCCWVM, lidocaine cream combing with valsalva maneuver; P, placebo; SC, standard care; VM, valsalva maneuver; SUCRA, surface under the cumulative ranking curve.

### 3.5 Comparison-adjusted funnel plot

**Figure 5:**
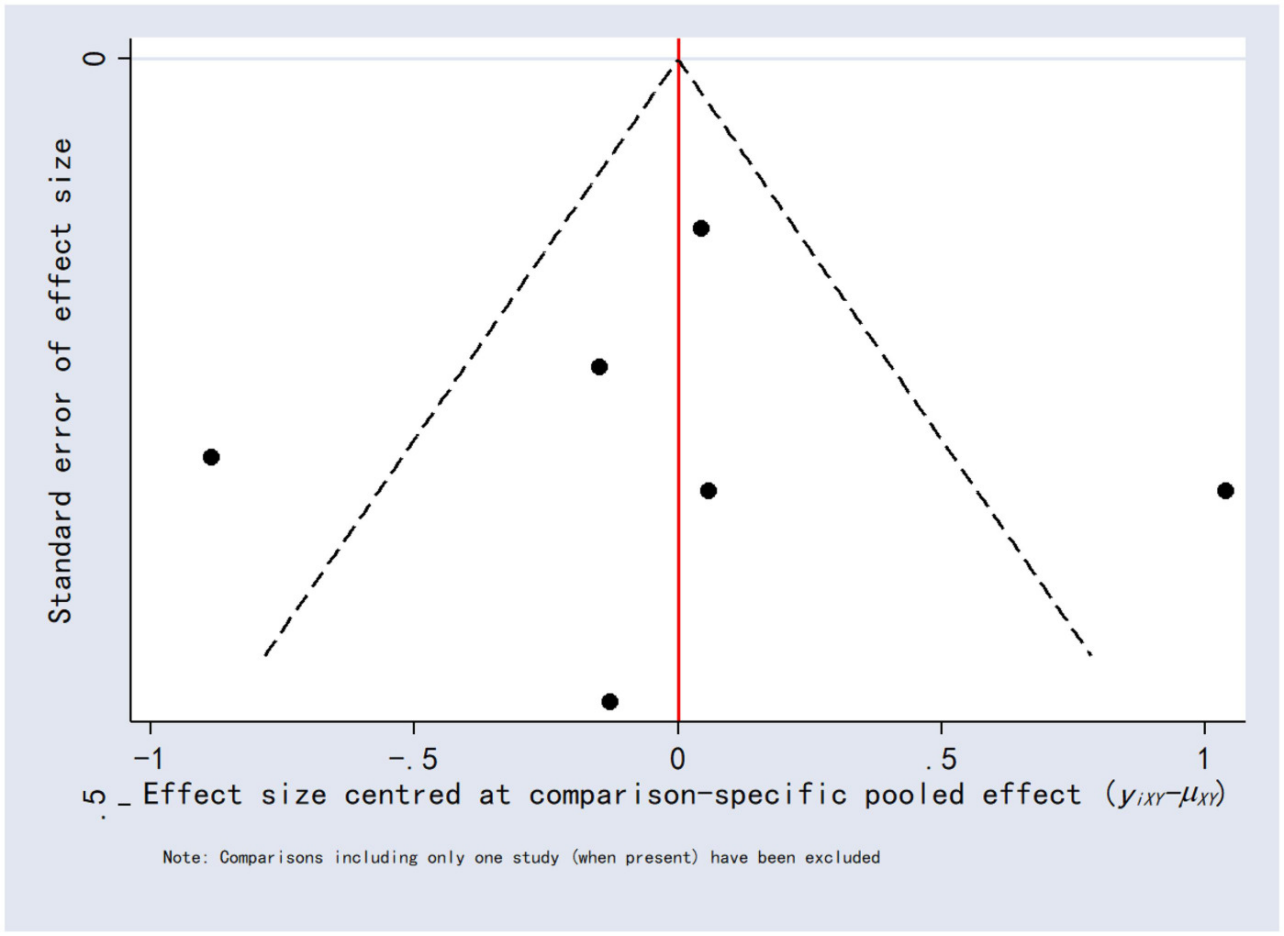
The funnel plot of included studies.

Publication bias was explored using a comparison-adjusted funnel plot. The results suggested potential publication bias or small-study effects (Figure 5). Given the limited number of included studies, this finding should be interpreted with caution.

### 3.6 Evaluation of evidence quality

The quality of evidence assessed using the CINeMA framework (Supplementary Table S3) indicated that the evidence for the intervention comparisons was rated as very low.

## 4 Discussion

Patients with a TIVAP frequently experience pain from non-invasive needle punctures over a long period of time, which carries a risk of causing long-term adverse physiological, psychological and behavioral reactions. Severe needle phobia may even lead them to avoid necessary diagnostic and therapeutic procedures. This study conducted a network meta-analysis to comprehensively compare the effectiveness of various interventions in alleviating non-coring needle puncture-related pain in TIVAP patients. The results showed that compared with placebo, standard care, cutaneous stimulation therapy, and cold spray, lidocaine cream combined with Valsalva maneuver significantly reduced the pain scores. Moreover, compared with standard care, lidocaine spray, lidocaine cream, and the Valsalva maneuver alone also reduced the pain scores. Compared with cutaneous stimulation therapy, lidocaine cream reduced pain scores. The probability ranking indicated that lidocaine cream combined with the Valsalva maneuver ranked highest, suggesting that this combination may be the optimal intervention for reducing non-coring needle puncture-related pain in TIVAP patients.

This network meta-analysis provides a comprehensive comparison of multiple interventions, offering more complete evidence to support clinical practice. The findings are consistent with several recent trials demonstrating that lidocaine-type local anesthetics can effectively alleviate pain during TIVAP puncture (22, 24, 26). The RCT conducted by Zhu et al. (26) confirmed that lidocaine spray significantly reduced the pain score (intervention group: 15.12 ± 6.61 mm vs. control group: 36.50 ± 18.79 mm). The analgesic effect of the Valsalva maneuver has also been supported by other studies, although its efficacy may be weaker than that of pharmacological interventions (23. This study is the first to directly compare the relative effectiveness of multiple interventions through a network meta-analysis, and to identify a potential synergistic effect of the combined intervention (lidocaine cream combing with Valsalva maneuver). Previous individual studies have mostly focused on isolated measures (e.g., lidocaine alone or cold therapy alone) and did not systematically evaluate combination strategies (23). Unlike some earlier findings, this study found that the effects of cold spray and cold therapy were limited (SUCRA probabilities of only 51.3% and 33.1% respectively), which may be related to their short duration of action and the high technical demands of application. Lidocaine blocks nerve terminal impulse conduction by inhibiting sodium ion channels, thereby rapidly interrupting pain sensation (26). The aerosol formulation can also produce a temporary cooling effect through volatile components (e.g., alcohol), further suppressing pain signals. The Valsalva maneuver is a physiological maneuver involving forced exhalation against a closed airway. Its analgesic effect during needle puncture has been observed in several clinical studies, although the underlying mechanisms are not fully established. Several hypotheses have been proposed: (1) it may activate the parasympathetic nervous system and modulate central pain processing via baroreceptor-mediated pathways; (2) it may serve as a distractor, redirecting the patient's attention away from the painful stimulus; (3) it might temporarily alter venous return and local venous pressure, which could theoretically influence tissue tension at the puncture site (23), but this remains speculative and lacks direct experimental evidence. Most likely, a combination of psychological and physiological factors contributes to the observed pain reduction. Currently, it is believed that psychological factors (attentional distraction) and physiological factors (autonomic nervous system modulation) may act together. Future mechanistic studies are needed to clarify how the Valsalva maneuver exerts its analgesic effect in this context. Lidocaine cream combined with the Valsalva maneuver simultaneously reduces tissue responses by pharmacologically blocking pain perception and through physiological regulation, potentially resulting in an additive or synergistic effect. Although the potential synergistic effect of the combined intervention is biologically plausible, it currently lacks direct evidence, and the ranking results of this study should be regarded as exploratory findings that require validation through specifically designed mechanistic studies and larger randomized controlled trials.

Lidocaine cream combined with the Valsalva maneuver can be considered a preferred option for alleviating pain during TIVAP puncture in clinical settings, especially for patients who are pain-sensitive or highly anxious. Lidocaine spray has a rapid onset (seconds to 1 min) and is easy to apply, making it suitable for outpatient, emergency, or situations where puncture must be completed quickly; however, attention should be paid to dose control and mucosal irritation, and excessive application or accidental eye contact should be avoided. Lidocaine cream needs to be applied 60–120 min in advance and covered with a dressing, making it appropriate for planned procedures (e.g., scheduled pre-chemotherapy maintenance puncture). Although more time-consuming, it provides a more stable anesthetic effect and is particularly suitable for patients who are pain-sensitive or have a high level of anxiety. The Valsalva maneuver is cost-free, non-pharmacological, and requires no waiting time, making it useful as an adjunct or as a standalone measure when drugs are not indicated. However, because it requires patient understanding and active cooperation, it is not suitable for uncooperative children, patients with cognitive impairment, or those with compromised cardiopulmonary function. Cold spray/cryotherapy can produce transient pain relief through an immediate cold sensation, but the effect is short-lived and low temperatures may cause discomfort or even skin injury; the specified distance and application time in the instructions should therefore be strictly followed. The combined intervention (lidocaine cream + Valsalva maneuver) may theoretically achieve a synergistic effect of peripheral anesthesia and central modulation; however, it requires both pre-application of the cream and patient cooperation, making the procedure relatively complex. Currently, it is supported by only one small-sample study and is not yet recommended as a routine first choice. The selection among these interventions can be adjusted according to patients' individual needs and preferences, thus enabling personalized pain management. In clinical practice, healthcare providers often need to choose appropriate pain relief measures when performing non-coring needle puncture in patients with a TIVAP.

## 5 Limitations

Although this network meta-analysis provides comprehensive evidence, several limitations should be acknowledged. First, the number of included studies is limited, and some have small sample sizes, which may affect the stability and reliability of the results. Second, differences in the specific implementation of interventions and in pain assessment methods across studies may have contributed to heterogeneity in the findings. Third, owing to the varying quality of the included studies, some studies carry a risk of bias, which may also affect the accuracy of the results. Finally, some interventions in the included studies (e.g., the Valsalva maneuver, cutaneous stimulation therapy) are difficult to blind for participants, which may lead to overestimation or underestimation of pain scores. As pain is a subjective endpoint, lack of blinding can introduce detection bias and performance bias. Therefore, the evidence for non-pharmacological or behavioral interventions should be interpreted more cautiously. Future studies should consider using sham procedures or placebo-controlled designs to improve blinding. Given the above limitations, especially the lack of direct comparisons and standardized outcome measures, the conclusions of this study should be regarded as exploratory findings. When referencing these results, clinicians should carefully select pain relief interventions by taking into account individual patient circumstances, procedural feasibility, and the limitations of the existing evidence. Before higher-quality evidence becomes available, the results of this study should not be used as a basis for strong clinical recommendations.

## 6 Conclusion

Based on the current evidence, lidocaine cream combined with the Valsalva maneuver had the highest probability of being the most effective among the evaluated interventions. However, the included randomized controlled trials were limited in number, the evidence network was sparse, the methodological quality of some studies was suboptimal, and direct head-to-head comparisons between different interventions were lacking. This finding should be regarded as an exploratory result derived from limited evidence and interpreted with caution. Therefore, strong clinical recommendations cannot be made at present. Larger, rigorously designed, and adequately blinded randomized controlled trials are needed to directly compare the most promising interventions (e.g., lidocaine cream combined with the Valsalva maneuver vs. lidocaine spray) and to employ standardized pain assessment tools together with clinically meaningful pain relief thresholds, in order to further validate these findings and guide clinical practice.

## Data Availability

The datasets presented in this study can be found in online repositories. The names of the repository/repositories and accession number(s) can be found in the article/Supplementary Material.
