# Supplementary material for: Effect of various interventions on relieving non-coring needle puncture-related pain in patients with totally implantable venous access port: a network meta-analysis of randomized control trials
Source: Front Surg. 2026 Jun 26;13:1839473. doi: 10.3389/fsurg.2026.1839473 (PMC13350041; doi:10.3389/fsurg.2026.1839473)
Supplement: Supplementary file 1 [file Datasheet1.docx]

| **Supplementary Table S1. Search strategy** | |
| --- | --- |
| Pubmed | ("totally implantable venous access port"[Title/Abstract] OR "TIVAP"[Title/Abstract] OR "TIAP"[Title/Abstract] OR "chemoport"[Title/Abstract] OR "port-a-cath"[Title/Abstract] OR "implantable port"[Title/Abstract]) AND ("non-coring needle"[Title/Abstract] OR "Huber needle"[Title/Abstract] OR "needle insertion"[Title/Abstract] OR "needle puncture"[Title/Abstract]) AND ("pain"[Title/Abstract] OR "analgesia"[Title/Abstract]) AND ("EMLA"[Title/Abstract] OR "lidocaine"[Title/Abstract] OR "cryotherapy"[Title/Abstract] OR "cold spray"[Title/Abstract] OR "Valsalva"[Title/Abstract] OR "cutaneous stimulation"[Title/Abstract] OR "massage"[Title/Abstract]) AND ("randomized controlled trial"[Publication Type] OR "RCT"[Title/Abstract] OR "random*"[Title/Abstract]) |
| Cochrane Library | (TIVAP OR TIAP OR chemoport OR port-a-cath OR "implantable port"):ti,ab,kw AND ("non-coring needle" OR "Huber needle" OR "needle insertion" OR "needle puncture"):ti,ab,kw AND (pain OR analgesia):ti,ab,kw AND (EMLA OR lidocaine OR cryotherapy OR "cold spray" OR Valsalva OR "cutaneous stimulation" OR massage):ti,ab,kw AND ("randomized controlled trial" OR RCT OR random*):ti,ab,kw |
| Embase | ('totally implantable venous access port'/exp OR 'tivap':ti,ab OR 'tiap':ti,ab OR 'chemoport':ti,ab OR 'port a cath':ti,ab OR 'implantable port':ti,ab) AND ('huber needle':ti,ab OR 'non coring needle':ti,ab OR 'needle insertion':ti,ab OR 'needle puncture':ti,ab) AND ('pain'/exp OR 'pain':ti,ab OR 'analgesia':ti,ab) AND ('lidocaine'/exp OR 'lidocaine':ti,ab OR 'emla':ti,ab OR 'cryotherapy'/exp OR 'cryotherapy':ti,ab OR 'cold spray':ti,ab OR 'valsalva maneuver'/exp OR 'valsalva':ti,ab OR 'cutaneous stimulation':ti,ab OR 'massage'/exp OR 'massage':ti,ab) AND ('randomized controlled trial'/exp OR 'randomized controlled trial':ti,ab OR 'rct':ti,ab OR 'random*':ti,ab) |
| CINAHL databases | ( (MH "Vascular Access Devices+") OR TI (TIVAP OR TIAP OR chemoport OR "port-a-cath" OR "implantable port") OR AB (TIVAP OR TIAP OR chemoport OR "port-a-cath" OR "implantable port") ) AND ( TI ("non-coring needle" OR "Huber needle" OR "needle insertion" OR "needle puncture") OR AB ("non-coring needle" OR "Huber needle" OR "needle insertion" OR "needle puncture") ) AND ( (MH "Pain+") OR TI (pain OR analgesia) OR AB (pain OR analgesia) ) AND ( (MH "Lidocaine") OR (MH "Anesthetics, Local+") OR TI (EMLA OR lidocaine OR cryotherapy OR "cold spray" OR Valsalva OR "cutaneous stimulation" OR massage) OR AB (EMLA OR lidocaine OR cryotherapy OR "cold spray" OR Valsalva OR "cutaneous stimulation" OR massage) ) AND ( (MH "Randomized Controlled Trials") OR (MH "Clinical Trials+") OR TI (RCT OR random* OR "randomized controlled trial") OR AB (RCT OR random* OR "randomized controlled trial") ) |
| CBM | #1: (完全植入式输液港 OR 植入式输液港 OR 输液港 OR 静脉输液港 OR TIVAP OR TIAP OR chemoport OR port-a-cath OR 植入式静脉输液装置 OR 静脉输液装置) AND (无损伤针 OR 非损伤针 OR 蝶翼无损伤针 OR Huber针 OR non-coring needle OR needle insertion OR needle puncture) #2: (疼痛 OR 镇痛 OR pain OR analgesia) #3: (利多卡因 OR EMLA OR 复方利多卡因 OR 局麻药 OR 冷喷剂 OR 冷疗 OR 冷敷 OR 瓦尔萨尔瓦动作 OR Valsalva OR 皮肤刺激 OR 按摩 OR 冷冻疗法) #4: (随机对照试验 OR 随机 OR RCT OR randomized OR 随机对照) #5：#1 AND #2 AND #3 AND #4 |
| CNKI | (SU=(输液港 + 植入式输液港 + 静脉输液港 + 完全植入式输液港 + 输液港座 + TIAP + TIVAP) OR TI=(输液港 + 植入式输液港 + TIAP + TIVAP) OR KY=(输液港 + TIAP + TIVAP)) AND (SU=(无损伤针 + 非损伤针 + 蝶翼无损伤针 + Huber针 + 'non-coring needle' + 'needle insertion') OR TI=(无损伤针 + 非损伤针 + 蝶翼无损伤针 + Huber针) OR KY=(无损伤针 + 蝶翼针 + Huber针)) AND SU=(疼痛 + 镇痛) AND (SU=(利多卡因 + 复方利多卡因 + EMLA + 冷喷 + 冷疗 + 冷敷 + 瓦尔萨尔瓦 + Valsalva + 皮肤刺激 + 按摩) OR TI=(利多卡因 + EMLA + Valsalva + 冷喷)) AND SU=(随机对照 + 随机 + RCT) |

| **Supplementary Material Table S2.The specific details of the intervention** | | |
| --- | --- | --- |
| Intervention | Original Research | Specific Operational Details |
| Lidocaine Cream | Liu 2018 | 2.5% lidocaine + 2.5% prilocaine, amount the size of a soybean (approximately 0.5 g), applied to an area the size of the injection port base, gently massaged after application, covered with clean plastic wrap, applied 1 hour before puncture |
|  | Shi 2023 | EMLA cream (2.5% lidocaine + 2.5% prilocaine), dose 1.5 g/10 cm², application area 2 cm × 2 cm, covered with 3M transparent dressing, left in place for 60–70 minutes, wiped off before puncture |
|  | Yin 2018 | EMLA cream, dose 2 mL, covered with a 6 cm × 7 cm transparent dressing |
|  | Shin 2020 | Lidocaine cream 1.0 g, application area <10 cm² diameter, covered with transparent dressing, applied 1 hour before puncture |
| Lidocaine Spray | Zhu 2023 | 10% lidocaine spray, approximately 10 cm from the puncture site, sprayed twice, 3 sprays each time, with an interval of 1–2 minutes; disinfection and puncture within 1–2 minutes after spraying |
| Cold Spray | Li 2022 | Aerosol - type skin cold spray, approximately 10 cm from the puncture site, pressed twice, each lasting 5 seconds (until the skin turns white), puncture within 30 seconds |
|  | Zhang 2019 | Aerosol - type skin cold spray, 10 cm from the puncture site, sprayed twice, 5 seconds each, puncture immediately |
| Cryotherapy | Shin 2020 | Cold gel ice pack (12 × 15 cm, 180 cm²), frozen in the freezer for 3 hours, applied 10 minutes before puncture for approximately 3 minutes |
| Cutaneous Stimulation Therapy | Shin 2020 | Apply pressure and slow circular massage stimulation with fingertips at a location 4 cm from the puncture site, covering an area of approximately 12 cm in diameter, for at least 2 minutes |
| Valsalva Maneuver | Shi 2023 | After a deep inhalation, close the glottis tightly and exhale forcefully, maintaining an intrathoracic pressure of approximately 40 mmHg for 10–15 seconds; the puncture is completed while the maneuver is maintained; the patient's total breath - holding time does not exceed 10 seconds |
| Lidocaine Cream + Valsalva Maneuver | Shi 2023 | EMLA cream (same as Shi 2023 protocol) + Valsalva maneuver (maintained during puncture) |
| Standard Care | Liu 2018, Shin 2020 | Direct puncture after routine disinfection, without any analgesic intervention |
| Placebo | Shi 2023, Yin 2018 | Shi 2023: Medical petrolatum (appearance and texture similar to EMLA), dose and procedure same as the EMLA group; Yin 2018: White moisturizing cream (appearance identical to EMLA), dose 2 mL |

| **Supplementary Table S3. Results of CiNeMa analysis** | | | | | | | | |
| --- | --- | --- | --- | --- | --- | --- | --- | --- |
| Comparison | Number of studies | Within-study bias | Reporting bias | Indirectness | Imprecision | Heterogeneity | Incoherence | Confidence rating |
| CS:LC | 2 | No concerns | Some concerns | No concerns | Major concerns | No concerns | No concerns | Very low |
| CST:CT | 1 | Some concerns | Some concerns | No concerns | Major concerns | No concerns | No concerns | Very low |
| CST:LC | 1 | Some concerns | Some concerns | No concerns | No concerns | Major concerns | No concerns | Very low |
| CST:SC | 1 | Some concerns | Some concerns | No concerns | Major concerns | No concerns | No concerns | Very low |
| CT:LC | 1 | Some concerns | Some concerns | No concerns | Major concerns | No concerns | No concerns | Very low |
| CT:SC | 1 | Some concerns | Some concerns | No concerns | Major concerns | No concerns | No concerns | Very low |
| LC:LCCWVM | 1 | Some concerns | Some concerns | No concerns | Major concerns | No concerns | No concerns | Very low |
| LC:P | 2 | Some concerns | Some concerns | No concerns | No concerns | Major concerns | No concerns | Very low |
| LC:SC | 2 | Some concerns | Some concerns | No concerns | No concerns | Major concerns | No concerns | Very low |
| LC:VM | 1 | Some concerns | Some concerns | No concerns | Major concerns | No concerns | No concerns | Very low |
| LCCWVM:P | 1 | Some concerns | Some concerns | No concerns | No concerns | Major concerns | No concerns | Very low |
| LCCWVM:VM | 1 | Some concerns | Some concerns | No concerns | Major concerns | No concerns | No concerns | Very low |
| LS:P | 1 | No concerns | Some concerns | No concerns | No concerns | Major concerns | No concerns | Very low |
| P:VM | 1 | Some concerns | Some concerns | No concerns | Major concerns | No concerns | No concerns | Very low |
| CS:CST | 0 | Some concerns | Some concerns | No concerns | Major concerns | No concerns | No concerns | Very low |
| CS:CT | 0 | Some concerns | Some concerns | No concerns | Major concerns | No concerns | No concerns | Very low |
| CS:LCCWVM | 0 | Some concerns | Some concerns | No concerns | Major concerns | No concerns | No concerns | Very low |
| CS:LS | 0 | No concerns | Some concerns | No concerns | Major concerns | No concerns | No concerns | Very low |
| CS:P | 0 | Some concerns | Some concerns | No concerns | Major concerns | No concerns | No concerns | Very low |
| CS:SC | 0 | Major concerns | Some concerns | No concerns | No concerns | Major concerns | No concerns | Very low |
| CS:VM | 0 | Some concerns | Some concerns | No concerns | Major concerns | No concerns | No concerns | Very low |
| CST:LCCWVM | 0 | Some concerns | Some concerns | No concerns | No concerns | Major concerns | No concerns | Very low |
| CST:LS | 0 | Some concerns | Some concerns | No concerns | No concerns | Major concerns | No concerns | Very low |
| CST:P | 0 | Some concerns | Some concerns | No concerns | Major concerns | No concerns | No concerns | Very low |
| CST:VM | 0 | Some concerns | Some concerns | No concerns | Major concerns | No concerns | No concerns | Very low |
| CT:LCCWVM | 0 | Some concerns | Some concerns | No concerns | Major concerns | No concerns | No concerns | Very low |
| CT:LS | 0 | Some concerns | Some concerns | No concerns | Major concerns | No concerns | No concerns | Very low |
| CT:P | 0 | Some concerns | Some concerns | No concerns | Major concerns | No concerns | No concerns | Very low |
| CT:VM | 0 | Some concerns | Some concerns | No concerns | Major concerns | No concerns | No concerns | Very low |
| LC:LS | 0 | Some concerns | Some concerns | No concerns | Major concerns | No concerns | No concerns | Very low |
| LCCWVM:LS | 0 | Some concerns | Some concerns | No concerns | Major concerns | No concerns | No concerns | Very low |
| LCCWVM:SC | 0 | Some concerns | Some concerns | No concerns | No concerns | Major concerns | No concerns | Very low |
| LS:SC | 0 | Some concerns | Some concerns | No concerns | No concerns | Major concerns | No concerns | Very low |
| LS:VM | 0 | Some concerns | Some concerns | No concerns | Major concerns | No concerns | No concerns | Very low |
| P:SC | 0 | Some concerns | Some concerns | No concerns | Major concerns | No concerns | No concerns | Very low |
| SC:VM | 0 | Some concerns | Some concerns | No concerns | No concerns | Major concerns | No concerns | Very low |
| lidocaine cream = LC, lidocaine spray = LS, cold spray = CS, cryotherapy = CT, cutaneous stimulation therapy = CST, lidocaine cream combing with valsalva maneuver = LCCWVM, placebo = P, standard care = SC, valsalva maneuver = VM. | | | | | | | | |
